# SARS2 simplified scores to estimate risk of hospitalization and death among patients with COVID-19

**DOI:** 10.1038/s41598-021-84603-0

**Published:** 2021-03-02

**Authors:** Hesam Dashti, Elise C. Roche, David William Bates, Samia Mora, Olga Demler

**Affiliations:** 1 Division of Preventive Medicine, Center for Lipid Metabolomics, Brigham and Women’s Hospital and Harvard Medical School, Boston, MA USA; 2 Broad Institute of MIT and Harvard, Cambridge, MA USA; 3 Division of Cardiovascular Medicine, Brigham and Women’s Hospital and Harvard Medical School, Boston, MA USA

**Keywords:** Risk factors, Computational biology and bioinformatics

## Abstract

Although models have been developed for predicting severity of COVID-19 from the medical history of patients, simplified models with good accuracy could be more practical. In this study, we examined utility of simpler models for estimating risk of hospitalization of patients with COVID-19 and mortality of these patients based on demographic characteristics (sex, age, race, median household income based on zip code) and smoking status of 12,347 patients who tested positive at Mass General Brigham centers. The corresponding electronic records were queried (02/26–07/14/2020) to construct derivation and validation cohorts. The derivation cohort was used to fit generalized linear models for estimating risk of hospitalization within 30 days of COVID-19 diagnosis and mortality within approximately 3 months for the hospitalized patients. In the validation cohort, the model resulted in c-statistics of 0.77 [95% CI 0.73–0.80] for hospitalization, and 0.84 [95% CI 0.74–0.94] for mortality among hospitalized patients. Higher risk was associated with older age, male sex, Black ethnicity, lower socioeconomic status, and current/past smoking status. The models can be applied to predict the absolute risks of hospitalization and mortality, and could aid in individualizing the decision making when detailed medical history of patients is not readily available.

## Introduction

On 29 August 2020, the Centers for Disease Control and Prevention (CDC) reported 291,985 new COVID-19 weekly cases in the U.S. that increased the total number of cases in the U.S. to 5,890,532 patients^1^. At the rise of the new surge in cases, designing models for predicting severity of COVID-19 illness is essential for public health strategies, as risk scores could enable allocations of limited medical resources and preparedness of healthcare facilities. The CDC reports age and medical comorbidities (e.g. chronic kidney disease, heart conditions, immunocompromised conditions, obesity, etc.) as leading risk factors of severe illness in patients with COVID-19^2^. The importance of these risk markers has been studied^3–16^, and significance of associations between severity of illness and different patient characteristics have been demonstrated. These studies reported association between higher age and severe illness, pre-pandemic health disparities and higher risk of severe COVID-19 outcomes in blacks and racial minorities^9,10,17^, importance of obesity^18^ and its impacts on infected children and adults^8,19,20^, increased severity of COVID-19 illness in immunodeficient patients^4,11^, the role of preexisting cardiovascular disease (CVD) and the use of cardiovascular medications^21–24^ on severity of outcomes, and effects of kidney and pulmonary diseases^3^. Smoking has also been associated with COVID-19 outcomes^25–28^. The largest COVID-19 cohort study on more than 10,000 COVID-19 related deaths in the UK^12^ indicated a few preexisting medical conditions were significantly associated with severity in non-white and low socioeconomical regions. In another study on mortality of patients with COVID-19 in intensive care units (ICU) in the Lombardy region of Italy, older age, male sex, and measured arterial oxygenation parameters on admission to ICU were independently associated with mortality, while they also identified risk factors from patients’ medical history (chronic obstructive pulmonary disease, hypercholesterolemia, and type 2 diabetes)^5^. In a similar study in the U.S., mortality rate of ICU patients was associated to older age, male sex, high body mass index, arterial oxygenation, liver and kidney disfunction on admission, and medical history of coronary artery disease and active cancer were independently associated with mortality^7^. The 4C mortality risk score^29^ introduced a model that uses age, sex, respiratory rate, peripheral oxygen saturation, Glasgow coma score, number of comorbidities, urea and C-reactive protein concentrations to estimate risk of mortality among hospitalized patients. Compared to previous models that utilized comprehensive lists of potential severity risk factors, the 4C Mortality Score could achieve high accuracy in the UK^29^.

In these studies, the list of investigated and recorded risk markers from the medical history of patients varied, which could be due to the complexity and challenges associated with extracting phenotypes from electronic health records (EHR) data^30–33^. In addition, recent surges in the number of patients with COVID-19 combined with the increasingly limited medical resources and challenges related to clinic- or hospital-based assessments highlight the need for simplified home-based prediction models to identify higher risk patients. Hence, simplified models that can accurately predict severity of the illness without the need of detailed examination of medical history could be more practical. In addition, patient characteristics on admission have been demonstrated to be strongly associated with the severity of illness, and the most common risk markers have been demographic variables. Therefore, we hypothesized that simplified models may provide a fast and reliable prediction of hospitalization of patients with COVID-19 and mortality among these patients. We examined this hypothesis using demographic variables and smoking status of patients tested positive for COVID-19 at Mass General Brigham (MGB) medical centers, Massachusetts, USA.

## Results

The examined population contained N = 12,347 patients tested positive for COVID-19 at MGB facilities. This population consists of 42.77% white, 15.91% black, 9.05% Hispanic, and 32.28% other/unknown races. Cumulative endpoints were 3401 hospitalized patients, from which 509 were deceased. Characteristics of these patients are shown in Table 1.

**Table 1:** Characteristics of N = 12,347 patients with COVID-19 from the Mass General Brigham electronic health records.

Characteristics	Outpatients N = 8946	Hospitalized N = 3401	Deceased inpatients N = 509	Total N = 12,347
Age (year), median (IQR)	42.0 (29.0–56.0)	62.0 (48.0–77.0)	78.0 (69.0–87.0)	47.0 (32.0–62.0)
Women, N (%)	4969 (55.5)	1616 (47.5)	215 (42.2)	6585 (53.3)
Race, N (%)				
White	3564 (39.8)	1717 (50.5)	322 (63.3)	5281 (42.8)
Black	1371 (15.3)	593 (17.4)	79 (15.5)	1964 (15.9)
Hispanic	920 (10.3)	197 (5.8)	27 (5.3)	1117 (9.0)
Other/not recorded	3091 (34.6)	894 (26.3)	81 (15.9)	3985 (32.3)
Median household income ($1000), median (IQR)	60.4 (53.3–86.2)	65.5 (53.3–94.8)	68.3 (55.0–98.6)	64.2 (53.3–90.5)
Smoking, N (%)				
Current	353 (3.9)	210 (6.2)	20 (3.9)	563 (4.6)
Former	1099 (12.3)	860 (25.3)	171 (33.6)	1959 (15.9)
Never	5133 (57.4)	1920 (56.5)	164 (32.2)	7053 (57.1)
Unknown	2361 (26.4)	411 (12.1)	154 (30.3)	2772 (22.5)

*IQR* interquartile range.

### Predicting risk of hospitalization

The fitted generalized linear model (GLM) in the derivation cohort of MGB’s non-employees (N = 10,496, 30.46% hospitalized) indicated significant associations between the examined variables and hospitalization (Table 2). The odds ratios (OR) indicated higher risks of hospitalization for older and male patients. Compared with white patients, Hispanic patients had lower risk of hospitalization while black patients were at the highest risk (test of trend p-value < 0.001). Although the OR of median household income was close to 1, higher income was associated with lower risk of hospitalization. Test for trend in smoking status was significant (p-value < 0.001) with current smokers at the highest risk, followed by former smokers, and finally non-smokers at a lower risk of hospitalization.

**Table 2:** Adjusted odds ratios of the examined variables for predicting risk of hospitalization among patients with COVID-19 and risk of mortality for the hospitalized patients (N = 10,496 patients; 30.46% hospitalized).

Variables	Median (IQR), N (%)	OR (95% CI)	P
**Hospitalization**			
Age (years)	48.0 (32.0–64.0)	1.53 (1.49–1.57)	< .001
Sex			
Female	5251 (50.03)	Ref.	Ref.
Male	5245 (49.97)	1.40 (1.28–1.54)	< .001
Race			< .001*
White	4444 (42.34)	Ref.	Ref.
Black	1472 (14.02)	1.30 (1.13–1.49)	< .001
Hispanic	974 (9.28)	0.58 (0.48–0.70)	< .001
Other/not recorded	3606 (34.36)	1.02 (0.91–1.15)	0.74
Median household income ($1000)	60.4 (53.3–86.2)	0.98 (0.96–0.99)	0.007
Smoking			< .001*
Current	489 (4.66)	1.44 (1.17–1.76)	< .001
Former	1772 (16.88)	1.22 (1.08–1.38)	0.002
Never	5715 (54.45)	Ref.	Ref.
Unknown	2520 (24.01)	0.53 (0.46–0.60)	< .001
**Mortality among hospitalized patients**			
Age (years)	63.0 (48.0–77.0)	1.92 (1.77–2.08)	< .001
Sex			
Female	1484 (46.42)	Ref.	Ref.
Male	1713 (53.58)	1.55 (1.24–1.93)	< .001
Race			< .001*
White	1627 (50.89)	Ref.	Ref.
Black	529 (16.55)	0.79 (0.57–1.09)	0.15
Hispanic	183 (5.72)	1.28 (0.77–2.06)	0.32
Other/not recorded	858 (26.84)	0.57 (0.42–0.77)	< .001
Median household income ($1000)	65.5 (53.3–94.8)	0.95 (0.92–0.99)	0.006
Smoking			< .001*
Current	191 (5.97)	1.42 (0.82–2.36)	0.19
Former	871 (27.24)	1.30 (1.00–1.68)	0.05
Never	1679 (52.52)	Ref.	Ref.
Unknown	456 (14.26)	4.87 (3.64–6.52)	< .001

Each variable shown was mutually adjusted for the other variables in the table. IQR: interquartile range. Medians, interquartile ratios, and percentages are reported on the derivation population. Odd ratios (OR) and the corresponding 95% confidence interval (CI) for age is reported per 10 years increment, and these values for median household income are shown per 10,000$.

*Test of trend p-value.

**Figure 1:**
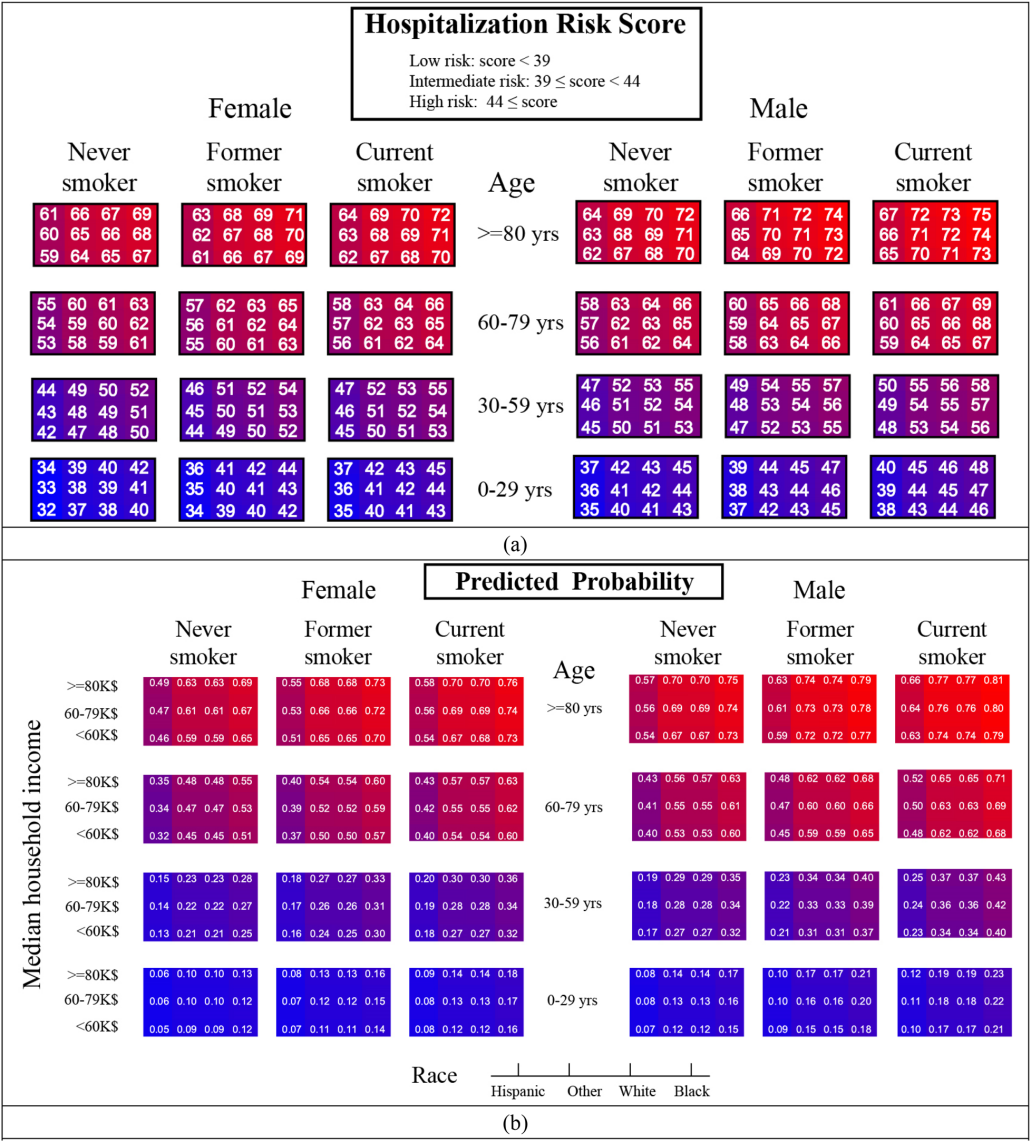
SARS2 scores heatmap for risk of hospitalization. (**a**) Scaled risk scores are divided into female patients (left panel) and male patients (right panel). The columns in each panel represent different smoking status. Every row indicates an age category. Each matrix shows the three median household incomes in rows, and columns of these matrices indicate four race/ethnic groups. The cells are colored from light-blue (lowest score: 0) to dark red (highest score: 68). The top panel indicates score ranges corresponding to low, intermediate, and high-risk groups. (**b**) Predicted risk probabilities. According to the US Census 2018, the lower median income cutoff ($60 K) represents the lower 62% of the population, and the high-income cutoff ($80 K) represents the top 15% of the population.

Examining this model in the validation cohort of MGB employees (N = 1851, 11.02% hospitalized) showed an area under the curve (AUC) of 0.77 [95% CI 0.73–0.80] (Supplementary Fig. 1a). The optimal predicted probability cutoff for discriminating between the two groups was 0.29, and the second optimal cutoff for identifying an intermediate risk group was 0.16. After applying these cutoffs on the MGB employees, the resulting receiver operating characteristic curve had an AUC of 0.73 [95% CI 0.70–0.76]. The model was well-calibrated in the validation cohort, based on the Hosmer–Lemeshow goodness of fit (GOF) test, p-value of 0.11. The GOF test was conducted after performing recalibration to adjust for different event rates in the derivation and validation cohorts. The corresponding calibration plot is shown in Supplementary Fig. 1b. After categorizing age (0–29, 30–59, 60–79, ≥ 80; years) and median household income (< 60, 60–80, ≥ 80; $1000), a GLM was fit on the derivation cohort and the model performed consistently with the main model (AUC in validation set: 0.75 [95% CI 0.71–0.78]). The ORs of this model were consistent with the main model (Supplementary Table 1). Heatmap of risk scores according to this categorization of age and median income is presented in Fig. 1a. Figure 1b shows the corresponding predicted probabilities of the categorized patient characteristics.

### Predicting mortality

The GLM model (Table 2) was fit to predict death among hospitalized patients with COVID-19 (N = 3401, 14.97% deceased). The AUC was 0.841 [95% CI 0.74–0.94] (Supplementary Fig. 1c). The optimal predicted probability cutoff point for distinguishing deceased vs. alive hospitalized patients was 0.10, and the second cutoff was 0.06. Applying these cutoffs resulted in AUC of 0.837 [95% CI 0.75–0.92]. Based on the Hosmer–Lemeshow GOF test, the model is well calibrated, p-value of 0.6 (Supplementary Fig. 1d).

### Sensitivity analyses

Effects of MGB’s change of policies in COVID-19 testing criteria before and after April 29, 2020 were considered. Two GLM models were trained on MGB non-employees who were tested for COVID-19 before (N = 6624, 33.57% hospitalized) and after (N = 3872, 25.13% hospitalized) April 29, 2020 that showed similar trends to the main model (Supplementary Table 2). Although the OR for median household incomes remained close to 1, the corresponding OR of the after April 29th cohort showed a different direction (OR 1.04 [95% CI 1.01–1.07], p-value 0.005) compared to the main model (OR 0.98 [95% CI 0.96–0.99], p-value 0.007). The ORs of the other characteristics (age, sex, race, and smoking) from the main model were confirmed in both before and after cohorts. We examined performance of the model predicting mortality of hospitalized patients with reference COVID-19 date before (N = 2379, 16.98% deceased) and after (N = 1022, 10.27% deceased) April 29th, 2020 (results not shown here). This analysis showed a good AUC of 0.76 [95% CI 0.72–0.81] for the model fit in the former group but evaluated in the latter group. For the patients tested after April 29th, the model showed an AUC of 0.81 [95% CI 0.78–0.83], when evaluated among patients tested prior to April 29, 2020. Effect sizes were consistent with the main model reported in Table 2, except income that was somewhat attenuated and no longer significant in the post April 29, 2020 subset (Supplementary Table 4).

### Risk groups

The optimal predicted probability cutoffs for hospitalization of patients with COVID-19 were 0.29 and 0.16, and 0.10 and 0.06 when predicting mortality among hospitalized patients. These cutoffs were used to define low, intermediate, and high-risk groups. The beta coefficients of the model were mapped according to 1 unit change that rescaled risk scores for hospitalization to 32–75 and 86–148 for mortality among hospitalized COVID-19 patients (Table 3). The rescaled cutoffs indicated high risk of hospitalization for patients with score ≥ 44, intermediate risk (39 ≤ score < 44), and low risk (score < 39). Similarly, high risk of mortality among hospitalized patients was assigned to scores ≥ 92, intermediate risk to 89 ≤ score < 92, and low risk patients have a score of less than 89. The prevalence of hospitalization within 30 days from COVID-19 diagnosis in the low, intermediate, and high risk groups were 2.75%, 8.47%, 22.85%, respectively. The incidence of mortality over approximately 3 months among hospitalized patients with COVID-19 diagnosis ranged from 0.92% in the low risk group to 4.44% and 26%, respectively, for the intermediate and high risk groups.

**Table 3:** SARS2 risk scores.

Characteristics	Hospitalization score	Mortality score
**Sex**		
Female	+ 10	+ 24
Male	+ 13	+ 27
**Age, years**		
0–29	0	0
30–59	+ 10	+ 24
60–79	+ 21	+ 37
≥ 80	+ 27	+ 46
**Race**		
Hispanic	+ 4	+ 25
White	+ 10	+ 24
Black	+ 12	+ 21
Other	+ 9	+ 17
**Socioeconomic status (median household income)**		
< $60 K	+ 10	+ 24
$60–$80 K	+ 9	+ 23
≥ $80 K	+ 8	+ 21
**Smoking status**		
Current	+ 13	+ 26
Ever	+ 12	+ 26
Never	+ 10	+ 24

The scores are defined for hospitalization within 30 days from COVID-19 diagnosis, and mortality within 74 days of the diagnosis.

Score categorization: Hospitalization: Low risk: score < 39, Intermediate risk: 39 ≤ score < 44, High risk: score ≥ 44. Mortality among hospitalized patients: Low risk: score < 89, Intermediate risk: 89 ≤ score < 92, High risk: score ≥ 92. The prevalence of hospitalization within 30 days from COVID-19 diagnosis in the low, intermediate, and high risk groups were 2.75%, 8.47%, 22.85%, respectively. The incidence of mortality over approximately 3 months among hospitalized patients with COVID-19 diagnosis ranged from zero 0.92% in the low-risk group, to 4.44% in the intermediate- and 26% in the high-risk groups. According to the US Census 2018, the lower median income cutoff ($60 K) represents the lower 62% of the population, and the high-income cutoff ($80 K) represents the top 15% of the population.

## Discussion

Currently, the U.S. is one of the epicenters of the pandemic with an increasing number of COVID-19 cases and mortality. The capability of predicting severity of COVID-19 illness in a fast and efficient manner would help healthcare workers to distinguish high risk patients. We utilized MGB EHR data of patients with COVID-19 to design simplified models for predicting hospitalization risk and also risk of mortality among hospitalized patients, where the model requires only demographic variables (age, sex, race, median household income) and smoking status of the patients. Testing the models on the validation cohorts showed high AUC (0.77 and 0.72 for hospitalization and mortality), and applying discrimination cutoffs for distinguishing patients with severe illness resulted in good AUCs as well. The Hosmer–Lemeshow GOF test resulted in p-values > 0.05 indicating good calibration of the SARS2 model.

Model performance characteristics such as AUC and Hosmer–Lemeshow GOF test calculated in set-aside validation cohorts indicated that the model has good discrimination and calibration, and performed well in the population of MGB patients. The odds ratios reported for our model are consistent with the currently available knowledge about association of severity of COVID-19 with demographic characteristics. This model is named “SARS2”, for its input variables: Sex, Age, Race, Socioeconomics status, Smoking status. The proposed SARS2 model is provided as a web interface for seamless calculation of the risk scores and risk categories (https://dashti.bwh.harvard.edu/sars2/).

In the main and the sensitivity analyses, Hispanic patients had a lower risk compared to white and black patients. Although these results align with the lower rate of hospitalized Hispanic patients in the current CDC reports (Hispanic: 22.9%, white: 31.7%, and black: 32.9%)^34^, analysis on the MGB’s EHR records showed 84.33% of Hispanic patients with COVID-19 are younger than 60 years. The younger age could explain the lower rate of hospitalization, and further investigations on Hispanic patients are needed. The derivation and validation cohorts are from patients tested positive for COVID-19 at MGB medical centers, and further validation of the models on other cohorts is required to establish generalizability beyond our data. Because of the complexity of EHR data, admission diagnoses and causes of death were not considered in this study. Therefore, although non-COVID-19 related admission rates dropped during the pandemic, some of our hospitalization and mortality endpoints may not be due to COVID-19 illness.

We would like to mention important strengths and limitations of this study. This study used registry data collected in large Boston-area hospitals; therefore it captures well medical records data for patients who were seen in this hospital system, but it is also limited to those who have an access to it. Simplicity of the proposed SARS2 model of risk of mortality allows medical professionals to use it in situations when rapid or home-based decisions must be made about the risk of severe outcome due to COVID-19. On the other hand, we intentionally did not use test results such as laboratory values and imaging data such as X-rays and history of comorbid conditions, which when available should be taken into account as well as the severity of symptoms at presentation. However, a parsimonious model such as SARS2 can be used when other information is not available or is not reliable.

In conclusion, the proposed SARS2 model for predicting hospitalization among patients with COVID-19, and mortality among those hospitalized patients is designed based on easily accessible risk markers (age, sex, race, median household income, and smoking status). The SARS2 risk score table can be used for rapid risk stratification and assessment of the severity of hospitalization and mortality risks of patients with COVID-19. The SARS2 scheme successfully identified COVID-19 patients who were at high risk of hospitalization (22.85% observed risk of hospitalization) as well as those at low risk of hospitalization (2.75%), allowing individualized selection of patients who may require closer monitoring or further evaluation. Furthermore, the SARS2 scheme provides a rapid estimate of risk of mortality that could be used immediately on arrival in the Emergency Department, when laboratory or radiological assessments are not available yet. It is well known that extraction of a valid history of medication-use, and diagnoses and preconditions is not always feasible or may result in further delays. Therefore, designing simplified, rapid, home-based models that can be used as prescreening at clinics or at home increases the practicality and efficiency of these models in healthcare facilities. Although there is a limited number of risk scores available for predicting hospitalization or death among patients with COVID-19, the SARS2 models presented here are on par with the c-statistics of more comprehensive models that for example predict mortality in the largest available COVID-19 cohort (average AUC of 0.77)^12^, or the 4C mortality score^29^ that in addition to demographic variables uses biomarkers (e.g. urea and C-reactive protein) and in-hospital measurements (e.g. respiratory and peripheral SO2) that resulted in an AUC of 0.77. Similarly, the survival model developed using cytokines, demographics and comorbidities on patients admitted to the Mount Sinai Health System in New York (AUC ranged from 0.65 to 0.76)^16^. The provided web interface for calculating SARS2 scores and estimated absolute risks enables reliable and rapid assessment of risks of hospitalization and mortality to be individualized.

## Methods

### Study population

**Figure 2:**
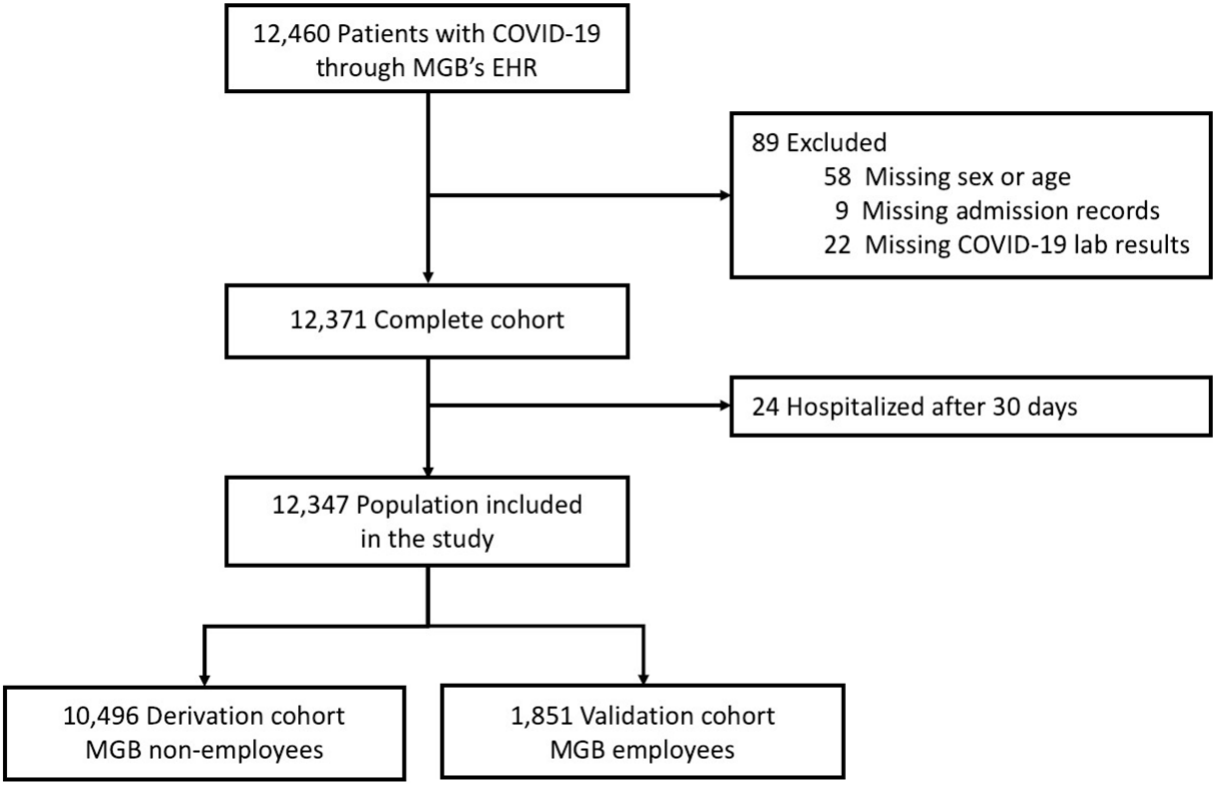
Study population diagram.

On 07/14/2020 a total of 12,460 individuals (outpatients and inpatients) have been diagnosed with COVID-19 at MGB medical centers. Demographic variables (age, sex, race, zip code), smoking status, hospital admission records, and COVID-19 lab results of these patients were queried from MGB’s EHR (Fig. 2). All data were obtained from Electronic Health Records Repository maintained by Mass General Brigham HealthCare Systems in full compliance with the Institutional Review Board (IRB) protocols and met all data access requirements. The study protocols have been reviewed and approved by the Partners Healthcare System IRB, and given the logistical complexities associated with the utilization of EHR data, informed consent was waived by the IRB for this study. Due to the use of EHR data, and the associated serious patient privacy concerns, the data utilized by this study is highly regulated and only released with appropriate IRB approval and under the most restrictive and carefully controlled conditions. As allowed by the IRB, summary statistics of the patient data have been included in the manuscript.

The COVID-19 lab results were dated within 03/04/2019–06/29/2020, and during this period, MGB employees working onsite underwent constant self-monitoring for symptoms and selective COVID-19 testing. The criteria for testing non-employees varied during the examined time interval; before April 29, 2020 symptomatic patients who were defined as high risk (e.g., age ≥70, severe chronic lung disease, sever heart disease, on immunocompromising medications, reside in counties with high number of cases) or of specific categories (e.g., pregnant ≥ 36 weeks, patients being discharged) were tested. However, a more relaxed criteria were applied after April 29, 2020 such that testing was not dependent on older age or preexisting medical conditions, and instead the criteria were defined based on symptoms (e.g., documented fever, cough, anosmia).

For every patient, the earliest positive (positive or presumptive positive) result of their COVID-19 tests was used as a reference date, and the time interval from these reference dates to the time of retrieving data for this study (07/14/2020) has a median follow-up of 84 days [95% IQR 69–96 days]. The EHR contained patients labeled as COVID-19 positive when their lab test results were positive/presumptive positive or patients were diagnosed with COVID-19 infection by the medical staff at MGB centers (COVID-19 ICD codes were used). Those without available COVID-19 lab test results were excluded from this study. The deceased flag and its corresponding date were retrieved from the EHR that indicated date of death among hospitalized patients within 74 days from the date of COVID-19 diagnosis (median date of death: 9 days [95% IQR 4–16 days]). Because of the waiting periods for receiving results of COVID-19 tests, hospital admission records dated between 7 days before until 30 days after patients reference date were queried from the EHR to identify hospitalized patients. Time to hospitalization ranged from − 7 to 29 days with median of 0 days, that reflects a positive COVID-19 diagnosis was a requirement for hospitalization in most cases. We note that the examined patient characteristics (age, sex, race, zip code, smoking status) are independent from time of events (hospitalization or death), and an ideal testing condition, with immediate availability of results, will not change associations between the examined characteristics and the events. Therefore, the events are considered as cumulative endpoints for the examined follow-up duration. We verified that outpatients had no record of admissions (more than 2 days) to MGB medical facilities during the period of − 7 to 30 days of follow-up.

In order to expand applications of the SARS2 models to more diverse regions in the U.S., we mapped patients’ primary zip codes to their median household incomes according to the U.S. Census 2018 data. These median household incomes were used as indicators of socioeconomic status of the patients. The EHR population contained 385 Asian, 18 Hawaiian, 30 American Indian, and 5 Dominicans that were considered as other races in the analysis.

MGB employees (validation cohort) and non-employees (derivation cohort) differed in their demographic characteristics (Supplementary Table 3) and also followed different COVID-19 testing criteria in the limited capacity setting. Presence of these differences between derivation and validation cohorts protects against over-optimism in estimating model performance characteristics and ensures robustness of the model. A logistic regression model (a generalized linear model with logit link (GLM)) was fit to predict hospitalization outcome. The same model was used for predicting mortality among the hospitalized patients.

To derive a model for predicting hospitalization of patients, we trained a GLM on demographic characteristics (sex, age, race, median household income), and smoking among non-employees (N = 10,496, 30.46% hospitalized) and validated the model on MGB employees (N = 1851, 11.02% hospitalized). Because mortality was recorded for inpatients, we examined the model performance for estimating mortality of the hospitalized patients (N = 3401, 14.97% deceased). In addition, because of the relatively lower rates of mortality among MGB employees, an average c-statistics of 5 iterations of validating the prediction model on randomly selected 20% of the hospitalized patients was also reported.

### Statistical methods

The EHR data were preprocessed using Python scripts. All variables (sex, age, race, median household income, and smoking status) were used in the R glm function to derive a multivariable model for predicting risk of hospitalization. In this model, linear associations with binomial distribution (logit link function) was used to distinguish between hospitalized vs. outpatient. The default glm convergence criteria on deviances was used to stop the iterations. The DeLong method was used to calculate confidence intervals for the c-statistics. The R coords function with Youden’s ‘best’ method was used to calculate the optimal cutoff points on the receiver operating characteristic curves. Model calibration was evaluated using Hosmer–Lemeshow goodness-of-fit (GOF) test (the R hoslem.test function) in the validation cohort, and the R plotCalibration function was used to plot the GOF calibration. A model was also fit after categorizing age (0–29, 30–59, 60–79, ≥ 80; years) and median household income (< 60, 60–80, ≥ 80; $1000). The cutoffs on the median household income correspond to (< 62%, 62–85%, ≥ 85%) of the US median household incomes, according to the 2018 Census 2018. The beta coefficients of this model were used to design a hospitalization heatmap. In order to enhance readability of the heatmap, risk scores were scaled to the minimum change in the coefficients. The p-values of the test of trend were reported in the derivation cohort. Because of the differences in testing criteria before and after April 29, 2020, a sensitivity analysis was conducted after dividing patients based on their corresponding reference dates. The same procedure as the main model were applied to the derivation and validation cohorts among patients tested before and after April 29, 2020. Additional sensitivity analysis was conducted on the population without discarding the 24 patients who have been hospitalized after the 30 days interval. In this analysis, these patients were considered as outpatients and a GLM was derived and examined.

The optimal cutoff for predicted probabilities was used to categorize patients into high risk category. Patients with estimated risks less than the above cutoff were then analyzed to calculate another optimal cutoff to define an intermediate risk category. Patients with estimated risk less than the second cutoff were reported as low risk. The same procedure was followed to group mortality risks of the hospitalized patients into low, intermediate, and high-risk groups.

A Python implementation of the risk prediction model with categorized age and income is hosted at our website for seamless public access (https://dashti.bwh.harvard.edu/sars2/).

## Supplementary Information

Supplementary Information

## References

[1] CDC. *COVID-19 cases in the U.S.*, https://www.cdc.gov/coronavirus/2019-ncov/cases-updates/cases-in-us.html (2020).

[2] CDC. *Assessing Risk Factors for Severe COVID-19 Illness*, https://www.cdc.gov/coronavirus/2019-ncov/covid-data/investigations-discovery/assessing-risk-factors.html (2020).

[3] Bajgain KT, Badal S, Bajgain BB, Santana MJ (2020). Prevalence of comorbidities among individuals with COVID-19: A rapid review of current literature. Am. J. Infect. Control.

[4] Gao Y, Chen Y, Liu M, Shi S, Tian J (2020). Impacts of immunosuppression and immunodeficiency on COVID-19: A systematic review and meta-analysis. J. Infect..

[5] Grasselli G (2020). Risk factors associated with mortality among patients with COVID-19 in intensive care units in Lombardy Italy. JAMA Internal Med..

[6] Grasselli G (2020). Baseline characteristics and outcomes of 1591 patients infected with SARS-CoV-2 admitted to ICUs of the Lombardy Region Italy. JAMA.

[7] Gupta SF (2020). Factors associated with death in critically ill patients with coronavirus disease 2019 in the US in the US. JAMA Internal Med..

[8] Kalligeros M (2020). Association of obesity with disease severity among patients with coronavirus disease 2019. Obesity.

[9] Pan, D. *et al.* The impact of ethnicity on clinical outcomes in COVID-19: A systematic review. *EClinicalMedicine* **23**. 10.1016/j.eclinm.2020.100404 (2020). 10.1016/j.eclinm.2020.100404 PMC7267805 32632416

[10] Price-Haywood EG, Burton J, Fort D, Seoane L (2020). Hospitalization and mortality among black patients and white patients with covid-19. N. Engl. J. Med..

[11] Siddiqi HK, Mehra MR (2020). COVID-19 illness in native and immunosuppressed states: A clinical-therapeutic staging proposal. J. Heart Lung Transplant..

[12] Williamson EJ (2020). OpenSAFELY: Factors associated with COVID-19 death in 17 million patients. Nature.

[13] Wu Z, McGoogan JM (2020). Characteristics of and important lessons from the coronavirus disease 2019 (COVID-19) outbreak in China: Summary of a report of 72,314 cases from the Chinese Center for disease control and prevention. JAMA.

[14] McMichael TM (2020). Epidemiology of Covid-19 in a long-term care facility in King County Washington. N. Engl. J. Med..

[15] Berlin DA, Gulick RM, Martinez FJ (2020). Severe Covid-19. N. Engl. J. Med..

[16] Del Valle DM (2020). An inflammatory cytokine signature predicts COVID-19 severity and survival. Nat. Med..

[17] Selden TM, Berdahl TA (2020). COVID-19 and racial/ethnic disparities in health risk, employment And Household Composition. Health Aff..

[18] Sattar, N., McInnes Iain, B. & McMurray John, J. V. Obesity is a risk factor for severe COVID-19 infection. *Circulation* **142**, 4–6. 10.1161/CIRCULATIONAHA.120.047659 (2020). 10.1161/CIRCULATIONAHA.120.047659 32320270

[19] Zachariah, P. *et al.* Epidemiology, clinical features, and disease severity in patients with coronavirus disease 2019 (COVID-19) in a children’s hospital in New York City, New York. *JAMA Pediatrics*, e202430-e202430. 10.1001/jamapediatrics.2020.2430 (2020). 10.1001/jamapediatrics.2020.2430 PMC7270880 32492092

[20] Lighter J (2020). Obesity in patients younger than 60 years is a risk factor for COVID-19 hospital admission. Clin. Infect. Dis..

[21] Aggarwal G (2020). Association of cardiovascular disease with coronavirus disease 2019 (COVID-19) severity: A meta-analysis. Curr. Probl. Cardiol..

[22] Mehra MR, Desai SS, Kuy S, Henry TD, Patel AN (2020). Cardiovascular disease, drug therapy, and mortality in Covid-19. N. Engl. J. Med..

[23] Reynolds HR (2020). Renin–angiotensin–aldosterone system inhibitors and risk of Covid-19. N. Engl. J. Med..

[24] Bandyopadhyay, D. *et al.* COVID-19 Pandemic: Cardiovascular complications and future implications. *Am. J. Cardiovasc. Drugs* 1–14. 10.1007/s40256-020-00420-2 (2020). 10.1007/s40256-020-00420-2 PMC7310596 32578167

[25] Lippi G, Henry BM (2020). Active smoking is not associated with severity of coronavirus disease 2019 (COVID-19). Eur. J. Intern. Med..

[26] Organization, W. H. Smoking and COVID-19, https://www.who.int/news-room/commentaries/detail/smoking-and-covid-19 (2020).

[27] Polosa, R. & Caci, G. COVID-19: counter-intuitive data on smoking prevalence and therapeutic implications for nicotine. *Intern. Emerg. Med*. 1–4. 10.1007/s11739-020-02361-9 (2020). 10.1007/s11739-020-02361-9 PMC7236870 32430652

[28] Rentsch, C. T. *et al.* Covid-19 testing, hospital admission, and intensive care among 2,026,227 United States veterans aged 54–75 years. *medRxiv*, 2020.2004.2009.20059964. 10.1101/2020.04.09.20059964 (2020).

[29] Knight SR (2020). Risk stratification of patients admitted to hospital with covid-19 using the ISARIC WHO Clinical Characterisation Protocol: Development and validation of the 4C Mortality Score. BMJ.

[30] Zhang, X. A. *et al.* Semantic integration of clinical laboratory tests from electronic health records for deep phenotyping and biomarker discovery. *NPJ Digit. Med.* **2**, 32. 10.1038/s41746-019-0110-4 (2019). 10.1038/s41746-019-0110-4 PMC6527418 31119199

[31] Xiao C, Choi E, Sun J (2018). Opportunities and challenges in developing deep learning models using electronic health records data: A systematic review. J. Am. Med. Inform. Assoc..

[32] Heisey-Grove D, Danehy L-N, Consolazio M, Lynch K, Mostashari F (2014). A national study of challenges to electronic health record adoption and meaningful use. Med. Care.

[33] Bayer R, Santelli J, Klitzman R (2015). New challenges for electronic health records: Confidentiality and access to sensitive health information about parents and adolescents. JAMA.

[34] CDC. *COVID-19 Laboratory-Confirmed Hospitalization*, https://gis.cdc.gov/grasp/COVIDNet/COVID19_5.html (2020).

