# Supplementary material for: SARS2 simplified scores to estimate risk of hospitalization and death among patients with COVID-19
Source: Sci Rep. 2021 Mar 2;11:4945. doi: 10.1038/s41598-021-84603-0 (PMC7925678; doi:10.1038/s41598-021-84603-0)
Supplement: Supplementary file 1 — Supplementary Information [file 41598_2021_84603_MOESM1_ESM.pdf]

SARS2 simplified scores to estimate risk of hospitalization and death among patients with COVID-19

Hesam Dashti[1,2], Elise C. Roche[1], David William Bates[1], Samia Mora[1,3*#], Olga Demler[1*]

[1]Center for Lipid Metabolomics, Division of Preventive Medicine, Brigham and Women's Hospital and Harvard Medical School, Boston, MA
[2]Broad Institute of MIT and Harvard, Cambridge, MA
[3] Division of Cardiovascular Medicine, Brigham and Women's Hospital and Harvard Medical School, Boston, MA
[*] Contributed equally
[#] Corresponding author: smora@bwh.harvard.edu

Supplementary material.

Supplementary Figure 1. Receiver operating characteristic (ROC) and calibration plots.
Supplementary Table 1. Adjusted odds ratios for predicting risk of hospitalization when age and median household income are categorized
Supplementary Table 2. Results of the sensitivity analysis on COVID-19 test date.
Supplementary Table 3. Characteristics of Mass General Brigham (MGB) employees and non-employees.
Supplementary Table 4. Results of the sensitivity analysis on COVID-19 test date predicting mortality among hospitalized patients.

Supplementary Figure 1. (a) Receiver operating characteristic (ROC) curve of the model for predicting hospitalization when the model applied on the validation cohort of Mass General Brigham (MGB) employees. (b) The Hosmer-Lemeshow goodness-of-fit (GOF) plot for the model predicting hospitalization on the validation population versus the observed hospitalizations (number of bins: 15, p-value: 0.11), demonstrating the model was well-calibrated. The higher rate of observed hospitalizations in the top bin compared to the mean predicted risks could be due in part to the use of MGB employees as the validation group who have better access to medical facilities. (c) ROC of the model for predicting mortality among hospitalized patients. (d) The Hosmer-Lemeshow GOF plot for the model predicting mortality on the hospitalized patients demonstrating the model was well-calibrated (number of bins: 15, p-value: 0.6).

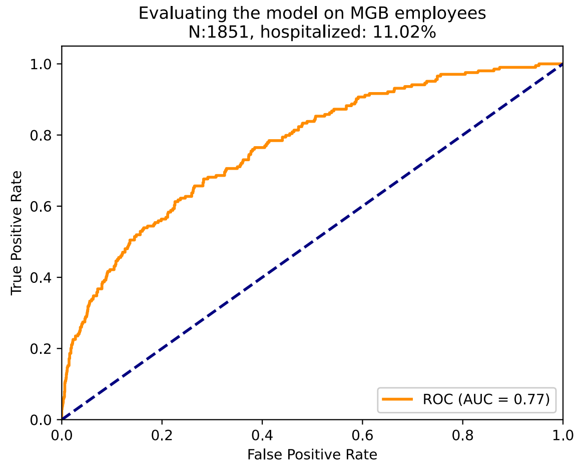

(a)

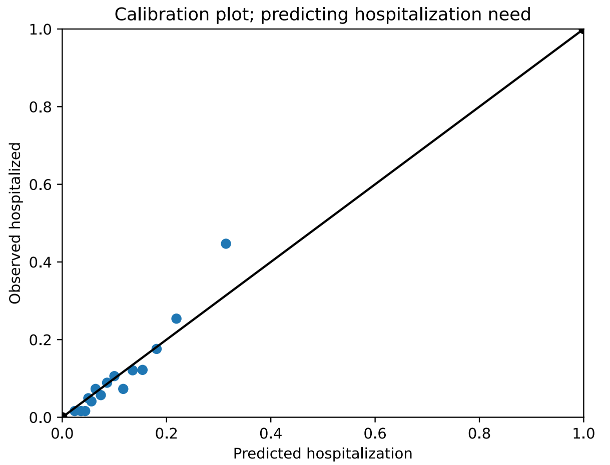

(b)

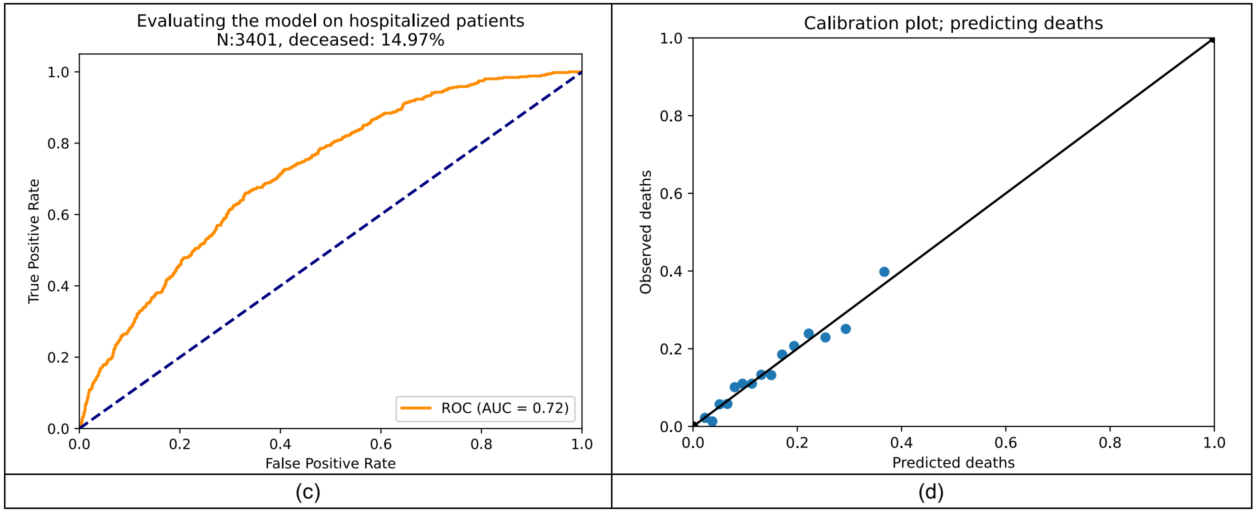
Evaluating the model on hospitalized patients
N:3401, deceased: 14.97%
True Positive Rate
False Positive Rate
ROC (AUC = 0.72)
Calibration plot; predicting deaths
Observed deaths
Predicted deaths

(c) (d)

Supplementary Table 1. Adjusted odds ratios for predicting hospitalization and mortality among hospitalized patients when age and median household income are categorized (N=10,496 patients; 30.46% hospitalized).

**Variables**	**Median (IQR), N (%)**	**OR (95% CI)**	**P**
**Hospitalization**			
Age (year)			<0.001*
0-29	2149 (20.47)	0.39 (0.33-0.45)	<.001
30-59	5040 (48.02)	ref	ref
60-79	2245 (21.39)	3.13 (2.80-3.50)	<.001
≥80	1062 (10.12)	5.61 (4.83-6.54)	<.001
Sex			
Female	5251 (50.03)	ref	ref
Male	5245 (49.97)	1.39 (1.27-1.53)	<.001
Race			<0.001*
White	4444 (42.34)	ref	ref
Black	1472 (14.02)	1.30 (1.13-1.49)	<.001
Hispanic	974 (9.28)	0.58 (0.48-0.69)	<.001
Other/Not recorded	3606 (34.36)	1.00 (0.89-1.12)	0.95
Median household income ($1000)			<0.001*
<60	4412 (42.04)	ref	ref
60-79	2936 (27.97)	0.94 (0.83-1.05)	0.25
≥80	3148 (29.99)	0.87 (0.78-0.98)	0.02
Smoking			<.001*
Current	489 (4.66)	1.42 (1.16-1.74)	<.001
Former	1772 (16.88)	1.25 (1.10-1.41)	<.001
Never	5715 (54.45)	ref	ref
Unknown	2520 (24.01)	0.52 (0.46-0.60)	<.001
**Mortality among hospitalized patients**			
Age (year)			<0.001*

0-29	210 (6.57)	0.09 (0.01-0.43)	0.02
30-59	1175 (36.75)	ref	ref
60-79	1144 (35.78)	3.76 (2.76-5.18)	<.001
≥80	668 (20.89)	9.86 (7.12-13.83)	<.001
Sex			
Female	1484 (46.42)	ref	ref
Male	1713 (53.58)	1.43 (1.15-1.78)	0.001
Race			<0.001*
White	1627 (50.89)	ref	ref
Black	529 (16.55)	0.77 (0.56-1.05)	0.1
Hispanic	183 (5.72)	1.19 (0.72-1.90)	0.48
Other/Not recorded	858 (26.84)	0.52 (0.38-0.71)	<.001
Median household income ($1000)			<0.001*
<60	1198 (37.47)	ref	ref
60-79	894 (27.96)	0.92 (0.70-1.21)	0.55
≥80	1105 (34.56)	0.80 (0.61-1.03)	0.09
Smoking			<.001*
Current	191 (5.97)	1.25 (0.72-2.05)	0.4
Former	871 (27.24)	1.32 (1.03-1.71)	0.03
Never	1679 (52.52)	ref	ref
Unknown	456 (14.26)	4.95 (3.72-6.58)	<.001

Odd ratios (OR) and the corresponding 95% confidence interval (CI) for each variable shown were mutually adjusted for the other variables in the table. IQR: interquartile range. Medians, interquartile ratios, and percentages are reported on the derivation population. * Test of trend p-value.

Supplementary Table 2. Results of the sensitivity analysis on COVID-19 test date.

	COVID-19 tested before April 29, 2020 (MGB non-employees N=6,624)			COVID-19 tested after April 29, 2020 (MGB non-employees N=3,872)			Main model (MGB non-employees N=10,496)		
**Variables**	**Median (IQR), N (%)**	**OR (95% CI)**	**P**	**Median (IQR), N (%)**	**OR (95% CI)**	**P**	**Median (IQR), N (%)**	**OR (95% CI)**	**P**
Age (years)	51.0 (35.0-65.0)	1.58 (1.53-1.63)	<.001	43.0 (28.0-61.0)	1.44 (1.38-1.50)	<.001	48.0 (32.0-64.0)	1.53 (1.49-1.57)	<.001
Sex									
Female	3285 (49.6)	ref	ref	1966 (50.8)	ref	ref	5251 (50.03)	ref	ref
Male	3339 (50.4)	1.46 (1.31-1.64)	<.001	1906 (49.2)	1.30 (1.10-1.52)	0.002	5245 (49.97)	1.40 (1.28-1.54)	<.001
Race			<.001*			<.001*			<.001*
White	2741 (41.4)	ref	Ref	1703 (44.0)	ref	ref	4444 (42.3)	ref	ref
Black	957 (14.4)	1.21 (1.02-1.43)	0.03	515 (13.3)	1.48 (1.17-1.87)	<.001	1472 (14.0)	1.30 (1.13-1.49)	<.001
Hispanic	627 (9.5)	0.64 (0.51-0.79)	<.001	347 (9.0)	0.40 (0.27-0.58)	<.001	974 (9.3)	0.58 (0.48-0.70)	<.001
Other/Not recorded	2299 (34.7)	1.03 (0.90-1.19)	0.66	1307 (33.8)	0.94 (0.76-1.15)	0.54	3606 (34.4)	1.02 (0.91-1.15)	0.74
Median household income ($1000)	60.4 (53.3-88.7)	0.95 (0.93-0.97)	<.001	60.4 (53.3-85.3)	1.04 (1.01-1.07)	0.005	60.4 (53.3-86.2)	0.98 (0.96-0.99)	0.007
Smoking			<.001*			<.001*			<.001*
Current	296 (4.5)	1.40 (1.08-1.81)	0.01	193 (5.0)	1.60 (1.15-2.22)	0.005	489 (4.7)	1.44 (1.17-1.76)	<.001
Former	1213 (18.3)	1.15 (0.99-1.33)	0.08	559 (14.4)	1.33 (1.07-1.65)	0.009	1772 (16.9)	1.22 (1.08-1.38)	0.002
Never	3666 (55.3)	ref	ref	2049 (52.9)	ref	ref	5715 (54.4)	ref	ref
Unknown	1449	0.51 (0.44-0.60)	<.001	1071 (27.7)	0.58 (0.47-0.73)	<.001	2520 (24.0)	0.53 (0.46-0.60)	<.001

Each variable shown was mutually adjusted for the other variables in the table. MGB: Mass General Brigham. IQR: interquartile range. Medians, interquartile ratios, and percentages are reported on the derivation population. Odd ratios (OR) and the corresponding 95% confidence interval (CI) for age is reported per 10 years increment, and these values for median household income are shown per 10,000$. * Test of trend p-value.

Supplementary Table 3. Characteristics of Mass General Brigham employees and non-employees.

**Characteristics**	**Non-employees N=10496**	**Employees N=1851**
Age (years), Median (IQR)	48.0 (32.0-64.0)	41.0 (30.0-54.0)
Women, N (%)	5251 (50.03)	1334 (71.88)
Race, N (%)		
White	4444 (42.34)	837 (45.10)
Black	1472 (14.02)	492 (26.51)
Hispanic	974 (9.28)	143 (7.70)
Other/Not recorded	3606 (34.36)	379 (20.42)
Median household income ($1000), Median (IQR)	60.4 (53.3-86.2)	70.2 (55.0-96.8)
Smoking, N (%)		
Current	489 (4.66)	63 (3.39)
Former	1772 (16.88)	253 (13.63)
Never	5715 (54.44)	1223 (65.89)
Unknown	2520 (24.01)	312 (16.81)

IQR: interquartile range

Supplementary Table 4. Results of the sensitivity analysis on COVID-19 test date predicting mortality among hospitalized patients.

	COVID-19 tested before April 29, 2020 N=2379			COVID-19 tested after April 29, 2020 N=1022			Main model in the derivation subset		
Variables	Median (IQR), N (%)	OR (95% CI)	P	Median (IQR), N (%)	OR (95% CI)	P	Median (IQR), N (%)	OR (95% CI)	P
Age (years)	63 (49-77)	2.07(1.89;2.28)	<.001	61 (43-76)	1.24(0.79;1.95)	.000	62 (48-77)	1.92 (1.77-2.08)	<.001
Female	1118 (47.0%)	ref	ref	498 (48.7%)	ref	ref	1616 (47.5%)	ref	ref
Male	1261 (53.0%)	1.75(1.36;2.26)	<.001	524 (51.3%)	1.6(1.38;1.86)	.346	1785 (52.5%)	1.55 (1.24-1.93)	<.001
White	1155 (48.5%)	ref	ref	562 (55.0%)	ref	ref	1717 (50.5%)	ref	ref
Black	409 (17.2%)	0.98(0.68;1.38)	.890	184 (18.0%)	0.52(0.24;1.04)	.079	593 (17.4%)	0.79 (0.57-1.09)	0.15
Hispanic	159 (6.7%)	1.18(0.68;1.96)	.547	38 (3.7%)	1.57(0.35;4.99)	.488	197 (5.8%)	1.28 (0.77-2.06)	0.32
Other/Not recorded	656 (27.6%)	0.57(0.4;0.81)	.002	238 (23.3%)	0.52(0.27;0.97)	.046	894 (26.3%)	0.57 (0.42-0.77)	<.001
Median household income ($1000)	6.5 (5.3-9.2)	0.94(0.9;0.98)	.002	6.8 (5.5-9.5)	1(0.94;1.07)	.897	6.5 (5.3-9.5)	0.95 (0.92-0.99)	0.006
Current	125 (5.3%)	1.46(0.77;2.6)	.222	75 (7.3%)	1.16(0.33;3.2)	.790	200 (5.9%)	1.42 (0.82-2.36)	0.19
Former	666 (28.0%)	1.14(0.85;1.52)	.390	261 (25.5%)	1.79(1.05;3.08)	.033	927 (27.3%)	1.30 (1.00-1.68)	0.05
Never	1281 (53.8%)	ref	ref	529 (51.8%)	ref	ref	1810 (53.2%)	ref	ref
Unknown	307 (12.9%)	4.91(3.51;6.89)	<.001	157 (15.4%)	5.51(3.06;10.02)	.000	464 (13.6%)	4.87 (3.64-6.52)	<.001

Each variable shown was mutually adjusted for the other variables in the table. MGB: Mass General Brigham. IQR: interquartile range. Medians, interquartile ratios, and percentages are reported on the derivation population. Odd ratios (OR) and the corresponding 95% confidence interval (CI) for age is reported per 10 years increment, and these values for median household income are shown per 10,000$. * Test of trend p-value.
